# Circadian Metabolomics in Time and Space

**DOI:** 10.3389/fnins.2017.00369

**Published:** 2017-07-11

**Authors:** Kenneth A. Dyar, Kristin L. Eckel-Mahan

**Affiliations:** ^1^Molecular Endocrinology, Institute for Diabetes and Obesity, Helmholtz Diabetes Center (HMGU) and German Center for Diabetes Research (DZD) Munich, Germany; ^2^Brown Foundation of Molecular Medicine for the Prevention of Human Diseases of McGovern Medical School, University of Texas Health Science Center at Houston Houston, TX, United States

**Keywords:** circadian, metabolism, metabolomics, metabolites, chronotherapy

## Abstract

Circadian rhythms are widely known to govern human health and disease, but specific pathogenic mechanisms linking circadian disruption to metabolic diseases are just beginning to come to light. This is thanks in part to the development and application of various “omics”-based tools in biology and medicine. Current high-throughput technologies allow for the simultaneous monitoring of multiple dynamic cellular events over time, ranging from gene expression to metabolite abundance and sub-cellular localization. These fundamental temporal and spatial perspectives have allowed for a more comprehensive understanding of how various dynamic cellular events and biochemical processes are related in health and disease. With advances in technology, metabolomics has become a more routine “omics” approach for studying metabolism, and “circadian metabolomics” (i.e., studying the 24-h metabolome) has recently been undertaken by several groups. To date, circadian metabolomes have been reported for human serum, saliva, breath, and urine, as well as tissues from several species under specific disease or mutagenesis conditions. Importantly, these studies have consistently revealed that 24-h rhythms are prevalent in almost every tissue and metabolic pathway. Furthermore, these circadian rhythms in tissue metabolism are ultimately linked to and directed by internal 24-h biological clocks. In this review, we will attempt to put these data-rich circadian metabolomics experiments into perspective to find out what they can tell us about metabolic health and disease, and what additional biomarker potential they may reveal.

## Introduction

The word “circadian” is derived from Latin, and literally means “around a day”. Thus, circadian rhythms oscillate with ~24-h periodicity. “Circadian metabolism” refers to 24-h oscillating biochemical changes in a given biological context (tissue, cell, sub-cellular compartment), and are readily apparent throughout human physiology. These include several systemic and tissue-specific metabolic pathways associated with activity/rest cycles, feeding/fasting rhythms, reproductive cycles, body temperature, and related rhythms of circulating hormones and metabolites. As such, time of day is a fundamental determinant of metabolic flux and pathway activity, and thus cannot be ignored. Metabolomics studies performed at a single time point provide only a static glimpse of metabolism relevant to that particular time point, and depending on the time selected and experimental condition, may easily miss the bigger picture. The same can be said for the particular tissue selected. High-resolution sampling of steady-state metabolite abundance over time and in space, i.e., combining multiple time points and different tissues throughout the circadian cycle, more faithfully captures the dynamic nature of metabolic pathways and inter-organ metabolite dynamics. This allows one to reconstruct a moving picture of circadian metabolism within or among tissues, and even postulate how related changes in metabolite concentrations reflect the actual metabolic pathway activity and dynamics within a tissue or compartment throughout the 24-h cycle. Subcellular resolution within metabolomics experiments likewise allows for a more complete picture, including a better inference of flux, since the transport of substrates into cells and across intracellular compartments are often rate-limiting steps regulating metabolic flux.

## Rhythmicity across tissues and organs

One major reason that rhythmic metabolites are important is that they provide key mechanisms by which clocks across the body communicate- whether in alignment or misalignment. Clocks in different cells and tissues respond to different synchronizing *zeitgebers* (“time-givers”), and proper communication between them is important for maintaining circadian homeostasis (Bass and Takahashi, 2010). Accordingly, cells isolated from organisms (i.e., mouse embryonic fibroblasts in culture) quickly become desynchronized in the absence of rhythmic synchronizing cues.

Often referred to as the “central pacemaker,” the suprachiasmatic nucleus of the brain is a light-responsive region of the anterior hypothalamus that allows for entrainment to the environment, and assists in the synchronization of tissues within the organism (Eastman et al., 1984; Akhtar et al., 2002; Welsh et al., 2004; Yoo et al., 2004; Reddy et al., 2007; Mendoza and Challet, 2009). The SCN maintains its light-responsiveness via melanopsin-containing neurons of the retinohypothalamic tract, which transmit light information from the retina to the SCN (Hattar et al., 2002; Ruby et al., 2002; Gooley et al., 2003). Circadian timing information regarding the light/dark cycle is then transmitted from the SCN to the periphery via sympathetic and parasympathetic neural circuits. Accordingly, lesion studies of the SCN in rodents reveal arrhythmic physiology.

One major SCN target is the pineal gland (Klein and Moore, 1979), and SCN-dependent neural activity results in the rhythmic release of melatonin, which is induced upon darkness in both nocturnal and diurnal species. Melatonin functions as a *zeitgeber*, binding to receptors located throughout the body, promoting oscillations in blood pressure, and buffering the body's immune response (Slominski et al., 2012; Carrillo-Vico et al., 2013). Other pathways of the sympathetic and parasympathetic nervous system also contribute to time-keeping in peripheral tissues. For example, hepatic rhythms in glucose output are a result of sympathetic innervation from the SCN to the liver via the paraventricular nucleus (PVN; Kalsbeek et al., 2004; Cailotto et al., 2005). The adrenal gland, which contributes to humoral regulation of peripheral clocks via the secretion of glucocorticoids, is also a key SCN-driven source of circadian communication to the peripheral organs. Glucocorticoids and their analogs can alter the phase of peripheral rhythms and even restore hepatic rhythmicity in the absence of a functioning SCN (Balsalobre et al., 2000; Reddy et al., 2007).

Indeed, several peripheral tissue circadian clocks seem to respond more robustly to non-neuronal *zeitgebers*, including circulating nutrients and hormones associated with feeding (Damiola et al., 2000; Stokkan et al., 2001; Vollmers et al., 2009). Specific examples of the robust response of peripheral clocks to nutrients include the restoration of hepatic rhythmicity in *Cry1/2* double knockout mice under time-restricted feeding (Vollmers et al., 2009), or the rescue of kidney and liver rhythmicity in forebrain/SCN-specific *Bmal1* knockout mice under constant darkness, again, by restricted feeding (Izumo et al., 2014). While the latter study suggests that not all peripheral tissues are entrained by feeding, there is clear evidence that circadian clocks in the liver, adipose, muscle, and kidney are all highly responsive to nutrients and/or the downstream signaling pathways that arise from acute changes in energy supply (i.e., high glucose). For example, liver expression of *Per2*, part of the negative feedback loop of the core circadian clock, is robustly driven by food intake (Zani et al., 2013), and liver *Per2* retains normal circadian oscillation even in the midst of hepatic circadian clock disruption (Kornmann et al., 2007; Lamia et al., 2008). Thus, relevant information regarding current energy substrate supply can be rapidly integrated into the circadian transcriptional program as it becomes available, serving to synchronize individual cells within a tissue and coordinating their functional response. One can easily imagine this would be particularly important for the major energy storing and energy consuming tissues.

**Figure 1:**
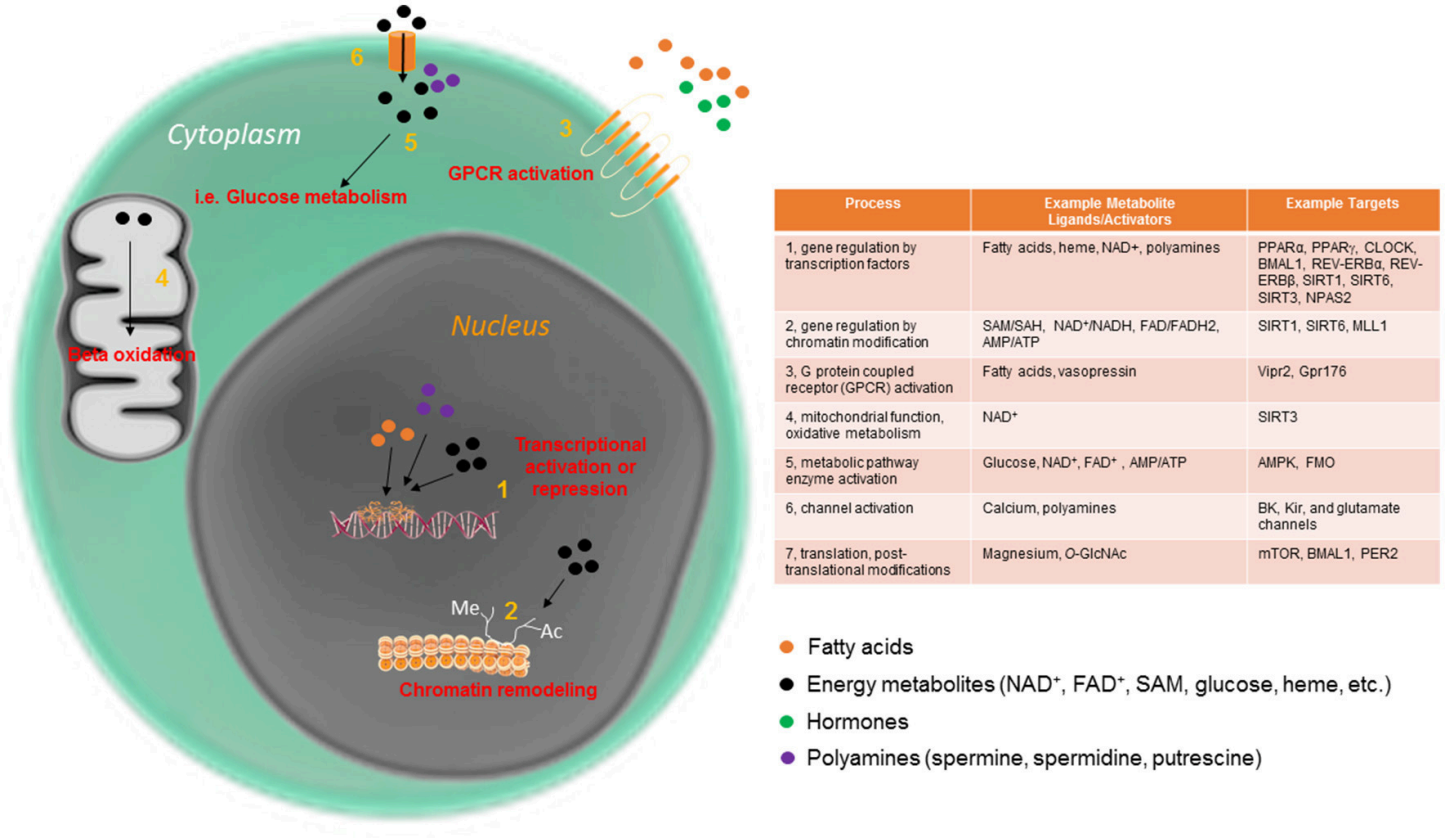
Circadian metabolites have pleiotropic mechanisms by which to maintain cellular rhythmicity. Metabolites are an essential part of the cellular circadian clock, and function intracellularly in a variety of ways, from direct metabolic pathway activation to mitochondrial and nuclear events.

Activation of various metabolic pathways within a cell is largely dependent on the cycling availability, sensitivity and transport of specific substrates. Thus, metabolic flux (the rate of flow/conversion of metabolites through a given metabolic pathway) is another rhythmic feature that groups individual cells within a given tissue into a functionally coherent unit, and reflects tissue-specific synchronization. Specific examples of metabolites that cycle, and that have also been revealed to be important for specific clock functions are discussed in subsequent sections (also see Figure 1 for examples).

## Maintenance of cellular circadian clocks

While there remains much to uncover, genomics studies have already revealed a wealth of information about the cellular determinants of circadian rhythmicity. Circadian rhythms of gene expression and metabolism are driven in part by circadian clock transcription factors (CLOCK and BMAL1 in mammals) that heterodimerize and activate target gene expression. Among these gene targets are their own negative regulators, the *Period* and *Cryptochrome* genes. Multiple peripheral loops of kinases, other transcription factors, phosphatases, and ubiquitin ligases, have been identified which keep the CLOCK:BMAL1 protein heterodimer and PER/CRY activity operating in a 24-h fashion (Partch et al., 2014). While these are largely outside the scope of this article, it is important to mention that disruption of these proteins and some of their ancillary regulators disrupts rhythmic metabolism in tissue-specific ways.

Much of the rhythmic activity of the core clock transcriptional feedback loop depends on interactions with other transcriptional regulators, as well as a large group of chromatin modifying enzymes (Aguilar-Arnal and Sassone-Corsi, 2015). For example, histone acetyl transferases, such as p300 and CREB-binding protein (CBP), can interact with the CLOCK:BMAL1 transcriptional complex (Etchegaray et al., 2003; Lee et al., 2015). Similarly, methyltransferases, such as the EZH2, WDR5 and the Mixed-Lineage Leukemia proteins have been shown to interact with the core clock proteins (Brown et al., 2005; Etchegaray et al., 2006; Katada and Sassone-Corsi, 2010). In short, rhythmic chromatin dynamics, much of which center on interactions with the clock proteins, are essential for driving rhythmic transcription and, ultimately, rhythms in metabolism (Koike et al., 2012; Menet et al., 2014). However, the transcriptional feedback loop supported by the CLOCK:BMAL1/PER:CRY system is not only maintained by ancillary proteins but also by small metabolites themselves, which activate specific components of this transcriptional feedback system as well as some of their associating factors.

Several small metabolites have been demonstrated to be important for 24-h rhythmicity in the cell across multiple organisms. In fact, to date, there are few cellular processes which have not been shown to be impinged upon by the circadian abundance or activity of small metabolites. Some of the many circadian processes directly regulated by metabolites are displayed in Figure 1. Many metabolites function by affecting rhythmic gene expression, either by activating proteins that directly bind to the core clock machinery or by acting at one or more of the many chromatin modifying enzymes involved in rhythmic gene expression. One well studied example is the metabolite nicotinamide adenine dinucleotide (NAD^+^), which as an activator of several NAD^+^-dependent sirtuin proteins, and controls the rhythmic circadian output at the level of gene expression of the CLOCK:BMAL1 transcriptional complex itself (Asher et al., 2008; Nakahata et al., 2008, 2009; Ramsey et al., 2009). In addition to nuclear activation of the sirtuins, rhythmic activation of SIRT3 by NAD^+^ in the mitochondria promotes oscillations in acetylation and activity of downstream enzymes important for mitochondrial oxidative function (Peek et al., 2013). Heme is another energy metabolite that controls circadian timekeeping at the cellular level. Heme is thought to bind directly to the circadian transcriptional repressors REV-ERBα and REV-ERBβ and thus influence their transcriptional activity (Raghuram et al., 2007; Yin et al., 2007). Heme is also considered to affect other clock components, including the NPAS2-BMAL1 heterodimer (Dioum et al., 2002; Ben-Shlomo et al., 2005). Nuclear receptors, including the REV-ERB proteins, are highly rhythmic in a tissue-specific way (Yang et al., 2006). This allows the cell to integrate and decode a diverse array of circadian changes in metabolite/ligand abundance directly into gene expression in a temporally and spatially precise manner. Many nuclear receptor ligands (some of which are diet-derived) are highly oscillatory (Sladek, 2011; Solt et al., 2011). Other oscillatory metabolites with specific effects on circadian metabolism include acetyl-CoA, which, via rhythmic sirtuin-dependent acetylation of the enzyme acetyl-CoA synthetase 1 (AceCS1), induces rhythmicity in fatty acid elongation (Sahar et al., 2014). DNA methylation, which depends on availability of S-adenosylmethionine (SAM) and S-adenosylhomocysteine (SAH), has also been reported to undergo circadian oscillations throughout the circadian cycle (Xia et al., 2015). While these serve as some of the better-characterized examples of metabolites that directly control the circadian clock, additional metabolites have been studied in this context. For example, polyamines (compounds with more than one primary amino [-NH2] group) have recently been shown to oscillate and provide feedback into the clock system (Zwighaft et al., 2015). Polyamines, which decrease with age, play important roles in the cell (Figure 1), binding to both DNA and proteins, and regulating processes as disparate as gene expression and ion channel function (Pegg, 2009). Rhythmicity in polyamine abundance is both clock- and feeding-dependent, with the rate-limiting enzyme in polyamine synthesis, ornithine decarboxylase (ODC), oscillating throughout the circadian cycle in response to BMAL1:CLOCK transactivation. Interestingly, polyamine depletion in cells and mice results in a longer circadian period due to impaired PER2:CRY1 protein interaction, whereas increasing polyamine content shortens circadian period (Zwighaft et al., 2015).

## How can high-throughput metabolomics help us understand circadian metabolism?

Just as genomics and transcriptomics studies have shed light on mechanisms regulating gene expression, global metabolomics experiments (if of sufficient temporal and spatial resolution) can help us understand circadian metabolism by revealing whole pathways of related metabolites that are changing in concert throughout the circadian cycle. Many good reviews already cover various technological aspects of metabolomics experiments (Zamboni et al., 2015).

While most of the studies reviewed here rely on datasets acquired by liquid chromatography/mass spectrometry (LC-MS), metabolite quantification has traditionally been performed using nuclear magnetic resonance spectroscopy (NMR) and MS. While considerably less sensitive than MS, NMR analysis can be performed with limited sample preparation, making it a preferable method in some situations. However, since sensitivity is lower than MS, higher initial sample volume is generally required.

Mass spectrometry, usually coupled with gas or liquid chromatography (GC-MS or LC-MS), can be performed in a high-throughput manner, and usually results in increased sensitivity. However, the more elaborate sample preparation may result in some metabolite losses. Also, the type of tissue under investigation may negatively impact ionization, and some poorly ionizing metabolites and metabolite classes may be underrepresented or missed altogether.

Overall, these should only be considered as very minor limitations, as ever more powerful MS-based metabolomics platforms have continued to evolve, and currently thousands of signals are normally collected and assigned a metabolite identity. However, many metabolites still cannot be assigned, as the specific metabolite identity may not have yet been described. Further complicating matters, mapping of many known metabolites to specific metabolic pathways (i.e., KEGG) is still incomplete, as the relatively slow pace of biological knowledge has not kept up with the accelerated pace of detection. While these “orphan” metabolites currently lack biological meaning, the breadth of information provided by circadian metabolomics studies can provide a unique context from which to predict their metabolic pathways, based on how they behave in relation to known metabolites. Thus, the size of the “known” metabolome in any given biological sample is likely to continue to rise substantially as more metabolite identities and their metabolic pathways are confirmed over time. Typically, assignment of LC-MS data to a particular metabolite is made based on the mass-to-charge value, retention time, fragmentation data, and isotopic detail. Various programs have been developed to assist with metabolite identification based on this information and have been described elsewhere (Zamboni et al., 2015).

While the specific number of metabolites identified in a typical metabolomics experiment is still currently some orders of magnitude smaller than analogous proteomics and transcriptomics experiments, coverage across various metabolite classes and metabolic pathways is usually sufficient to gain important biological insight. For example, comparative statistics and bioinformatics analyses, including metabolic pathway enrichment analysis, time-series (ANOVA), circadian analysis (e.g., JTK_Cycle) provide valuable starting points for investigation.

Spatial resolution is currently a major limitation for many circadian metabolomics studies. Metabolites can serve very different physiological roles depending on their intracellular localization. They can move across various intracellular compartments, either by diffusion or active transport, or even remain in different intracellular pools. While most circadian metabolomics studies published to date have largely ignored relevant information regarding subcellular localization of metabolites (i.e., mitochondrial vs. cytosolic pools), recent technological improvements are certain to change this trend. Subcellular fractionation and organelle purification prior to performing metabolomics is already used to help obtain a slightly clearer picture of intracellular metabolite distribution, but the time-consuming steps involved likely introduce additional confounders. On the other hand, matrix-assisted laser desorption/ionization coupled to Imaging Mass Spectrometry (MALDI-IMS) is already proving to be a very promising technology for mapping distribution of metabolites and proteins within tissue sections *in situ* (Norris and Caprioli, 2013; Ly et al., 2016).

We previously mentioned that relative changes in pathway activity and circadian dynamics may be inferred based on the relative changes in tissue steady-state metabolite levels. However, it is important to stress that these types of analyses can serve only as imperfect surrogates, and not as substitutes for actual metabolic flux analysis. Targeted pulse-chase biochemical and functional assays are still necessary for proper validation of these circadian dynamics. However, this may all soon change, as the increasing development and availability of specific fluorescent probes for monitoring 24-h metabolic flux *in vivo* are poised to provide additional essential tools in the near future.

## What have we learned so far from circadian metabolomics studies?

To date, several circadian metabolomes from a variety of organisms and tissues have been published. For obvious practical reasons related to collecting sufficient numbers of samples under tightly controlled genetic or environmental conditions, most circadian metabolome studies have been performed on rodent tissues. However, several high-resolution human circadian metabolomes have also been reported, which biologists and clinicians can already utilize for hypothesis generation/validation and biomarker discovery. For example, several human serum metabolomes have provided high-resolution snapshots of robustly changing metabolic pathways across the circadian cycle (Minami et al., 2009; Dallmann et al., 2012; Kasukawa et al., 2012; Davies et al., 2014; Giskeodegard et al., 2015). Importantly, these studies have yielded interesting information that must be interpreted in the context of the experimental paradigm. Specifically, Dallman et al. (Zamboni et al., 2015) provided the first global circadian metabolome from humans, in which approximately 15% of the metabolome in plasma or saliva was shown to oscillate throughout the circadian cycle under constant conditions of sleep deprivation and isocaloric hourly feeding. As feeding is a strong driver of peripheral circadian rhythms, it may be expected that a much higher percentage of metabolites would be found to oscillate throughout a more typical 24-h day, with normal eating and sleeping rhythms. Indeed, subsequent studies have revealed that a majority of detected metabolites show circadian rhythmicity when the sleep/wake cycle and energy intake patterns are rhythmic (Davies et al., 2014). Specifically, human plasma samples collected every 2 h for 48 h revealed that a majority of metabolites oscillate under controlled laboratory conditions (environmental light, sleep, meals, and posture) during a complete 24-h wake/sleep cycle. Furthermore, acute sleep deprivation over the following 24 h alters only a small percentage of oscillating metabolites (under 20%). Interestingly, metabolites elevated by sleep deprivation, including serotonin, tryptophan and taurine, may explain why acute sleep deprivation has been shown to have an antidepressive effect (Voderholzer, 2003). Similar studies involving chronic sleep deprivation have not yet been performed. Thus, for humans, most circulating metabolites display rhythmic diurnal oscillation under normal physiological conditions. This rhythmicity likely helps to coherently communicate time of day to tissues throughout the body, maintains tissue-specific synchronization of circadian clocks, and promotes efficient temporal gating of circadian metabolic pathways.

In a similar study comparing the effects of sleep deprivation on a smaller group of metabolites obtained from human urine, 22% of metabolites were observed to be rhythmic, either under normal or sleep-deprived conditions (Giskeodegard et al., 2015). Approximately half of the metabolites were altered by acute 24-h sleep deprivation, with similar numbers being increased or decreased as a result of sleep deprivation. Similar to results obtained from human plasma, metabolites related to tryptophan metabolism and taurine were also increased in urine as a result of sleep deprivation. Consistent with human 40-h sleep deprivation results (Dallmann et al., 2012), the tryptophan metabolite 3-indoxyl sulfate (which is produced from tryptophan by gut bacteria and hepatic cytochrome P450 enzymes) was also significantly elevated after acute sleep deprivation.

Circadian metabolomics studies performed on rodent tissues have also revealed a high degree of circadian oscillation among metabolites, largely reflecting diurnal rhythms of local tissue metabolism. For example, numerous amino acids oscillate in the liver throughout the circadian cycle (Eckel-Mahan et al., 2012), with pathways involved in lysine and glutamate metabolism particularly rhythmic. In addition, nicotinamide-related metabolites, including nicotinamide adenine dinucleotide (NAD^+^), oscillate robustly in mouse liver (Eckel-Mahan et al., 2012, 2013; Masri et al., 2014). As previously mentioned, NAD^+^ has substantial influence over cell rhythmicity as an activator of NAD^+^-dependent sirtuins and their interactions with the clock transcriptional machinery (Asher et al., 2008; Nakahata et al., 2008, 2009; Ramsey et al., 2009; Masri et al., 2014). Some lipid species are also highly rhythmic in livers, and even persist in livers of clock-disrupted *Per1/2* null mice, likely due to the maintenance of diurnal expression of *Ppar*α, a master regulator of lipid metabolism (Adamovich et al., 2014). In this study, feeding time again emerged as a particularly strong driver for some oscillating liver metabolites. *Per1/2* null mice showed arrhythmic feeding patterns and a drastically shifted diurnal cycle of triglyceride accumulation. However, restricting food access to exclusively the dark phase was sufficient to consolidate peak diurnal triglyceride accumulation in *Per1/2* null mice to around ZT12, as occurred in wildtype mice (Adamovich et al., 2014), suggesting that rhythmic liver triglyceride accumulation is driven by systemic feeding/fasting cycles independently of a functional hepatocyte circadian clock.

Uncovering the molecular mechanisms that drive circadian metabolite oscillations in tissues, and understanding the extent to which they are linked to tissue-specific circadian clocks remains an important and active area of research. A few studies have examined how the core circadian clock regulates circadian oscillations of specific metabolites (Eckel-Mahan et al., 2013; Dyar et al., 2014; Aviram et al., 2016). For example, while muscle-specific *Bmal1* knockout mice (mKO) display reduced muscle insulin sensitivity and impaired muscle glucose oxidation compared to wildtype littermates, robust circadian cycles of most glycolytic intermediates are apparently normal (Dyar et al., 2014). However, mKO muscles show a large general accumulation of sugar metabolites from several pathways directly linked to glycolysis, including the pentose phosphate, polyol, and glucuronic acid pathways. This suggests that muscle clock disruption uncouples glycolysis from glucose oxidation, and diverts glycolytic intermediates toward alternative metabolic fates. Interestingly, many of these metabolites were found to be increased predominantly around the transition from the fasting+rest phase to the feeding+activity phase, when glycolytic flux is increased, and glucose becomes the predominant fuel source for skeletal muscle. In conditional and inducible muscle-specific *Bmal1* knockout mouse models, the authors noted impaired circadian oscillation of PDP1 mRNA and protein, the main activator of the pyruvate dehydrogenase complex (PDH), as well as persistent circadian oscillation of PDK4 mRNA and protein, the main inhibitor of PDH. Likewise, diurnal PDH activity was found to be impaired, especially around the transition from the fasting+rest phase to the feeding+activity phase. Thus, BMAL1-driven rhythms are important for muscle insulin sensitivity and for maintaining metabolic flexibility by promoting glucose oxidation during the fasting to feeding transition. Similar to the loss of BMAL1, lack of its heterodimer, CLOCK, results in profound disruption of the hepatic circadian clock (Eckel-Mahan et al., 2012). *Clock*-deficient livers show impaired circadian pyrimidine metabolism due to loss of CLOCK-dependent transcriptional activation of specific enzymes, such as uridine phosphorylase. In addition, circadian carbohydrate metabolism is considerably flattened, while many feeding-related metabolites peak prior to the onset of the dark phase, consistent with an advanced onset of food intake. The circadian metabolomes of these and other circadian models can be found at the website for CircadiOmics (http://circadiomics.igb.uci.edu/). (For data integration and analysis with the corresponding transcriptomes also see Patel et al., 2012, 2015).

Another open issue based on these results is the extent to which rhythmicity in cell metabolism is cell autonomous. To help answer this question, metabolite profiling was recently performed on mouse liver as well as a cell autonomous system [human U2 OS (bone osteosarcoma) cells] with a 1–2 h resolution over the course of 48-h (Krishnaiah et al., 2017). Similar to prior studies, approximately half of the detected metabolites from liver were determined as rhythmic following circadian synchronization. Enrichment was observed for metabolites associated with nucleotide and amino acid metabolism, but methylation pathway-associated metabolites were also observed to by highly rhythmic. Interestingly, in both hepatocytes and autonomous osteosarcoma cells, metabolite oscillations, in terms of percent of detected metabolites, exceeded the percent of transcript oscillations, suggesting an increase in oscillatory function in the cell from transcription to metabolism. Cell-autonomous osteosarcoma cells showed less rhythmicity at the level of individual metabolites than liver (which could also be due to their transformed state); however, metabolites that were observed to be rhythmic in both cell types included several involved in epigenetic regulation. Loss of the circadian proteins BMAL1 generally decreased the amplitude of metabolite rhythms, while CRY1-depletion actually induced 8-h oscillation patterns for a number of metabolites. Thus, rhythmicity of many metabolites is a clock-driven and cell autonomous process (Krishnaiah et al., 2017).

In addition to global and tissue-specific genetic perturbation of clock genes, and restricting access to food, high fat diet (HFD) is another common experimental model used to elicit circadian clock disruption. Circadian metabolomics studies performed under different diets (i.e., chow vs. HFD) suggest that the serum circadian metabolome may be more susceptible to disruption by nutrient challenge than other tissues (Abbondante et al., 2016). Serum metabolites are profoundly affected by HFD, with over half of metabolites changing significantly at some circadian time point following prolonged high fat diet feeding. Similar to studies looking at oscillating metabolites in humans during relatively normal sleeping and feeding behaviors (Davies et al., 2014), mice engaged in *ad libitum* (rhythmic) feeding on a typical chow diet reveal metabolite oscillations in approximately half of the serum metabolome. However, unlike the liver, where many metabolite and transcript oscillations persist under HFD feeding (Eckel-Mahan et al., 2013), oscillating serum metabolites are highly prone to disruption by nutrient challenge, with the majority of oscillating metabolites eliminated after HFD in spite of rhythmic eating patterns (Eckel-Mahan et al., 2013; Abbondante et al., 2016). A profound contributor to this loss of oscillation is the ablation of rhythmic lipid metabolites. Similar to the circadian imbalance of some circadian lipid metabolites under conditions of *Bmal1* deficiency in adipose tissue (Paschos et al., 2012), it is worth speculating that hypothalamic sensing of fatty acids under conditions of HFD feeding is temporally impaired. This loss of circadian oscillations in the circulation under HFD reveals that not only is there unlikely to be temporal coherence across tissues that release metabolites into the blood, but also that the circulation is unable to provide rhythmic information from one tissue to another under conditions of nutrient challenge. This has tremendous implications as to the extent to which diet may misalign circadian clocks in the body, a process which is thought to be disadvantageous in terms of energy balance (Froy, 2010; Roenneberg et al., 2012; Asher and Sassone-Corsi, 2015; Ribas-Latre and Eckel-Mahan, 2016). Further circadian analyses of additional tissue metabolomes under conditions of nutrient challenge is likely to reveal the extent to which such misalignment across tissues might occur.

Recent studies suggest that many metabolites oscillate according to the organelle in which they reside. For example, a recent study looking at circadian lipidomics in the mitochondria and nucleus revealed that while many lipids oscillated in both compartments, some lipid species oscillate distinctly based on their location (Aviram et al., 2016). Nuclear lipid species tend to peak early in the light phase (ZT2-4) while most of the oscillating lipid species within the mitochondria peaked between ZT10-14, in line with the onset of the dark period. Feeding is again an important modulator of oscillating lipids in both compartments, with dark phase restricted feeding inducing more nuclear lipid oscillations, while decreasing the number of oscillating lipid species in the mitochondria. While the direct functional relevance of these differentially oscillating species has not been fully explored, it is likely that they reflect the unique demands associated with each compartment during different times of the day.

**Figure 2:**
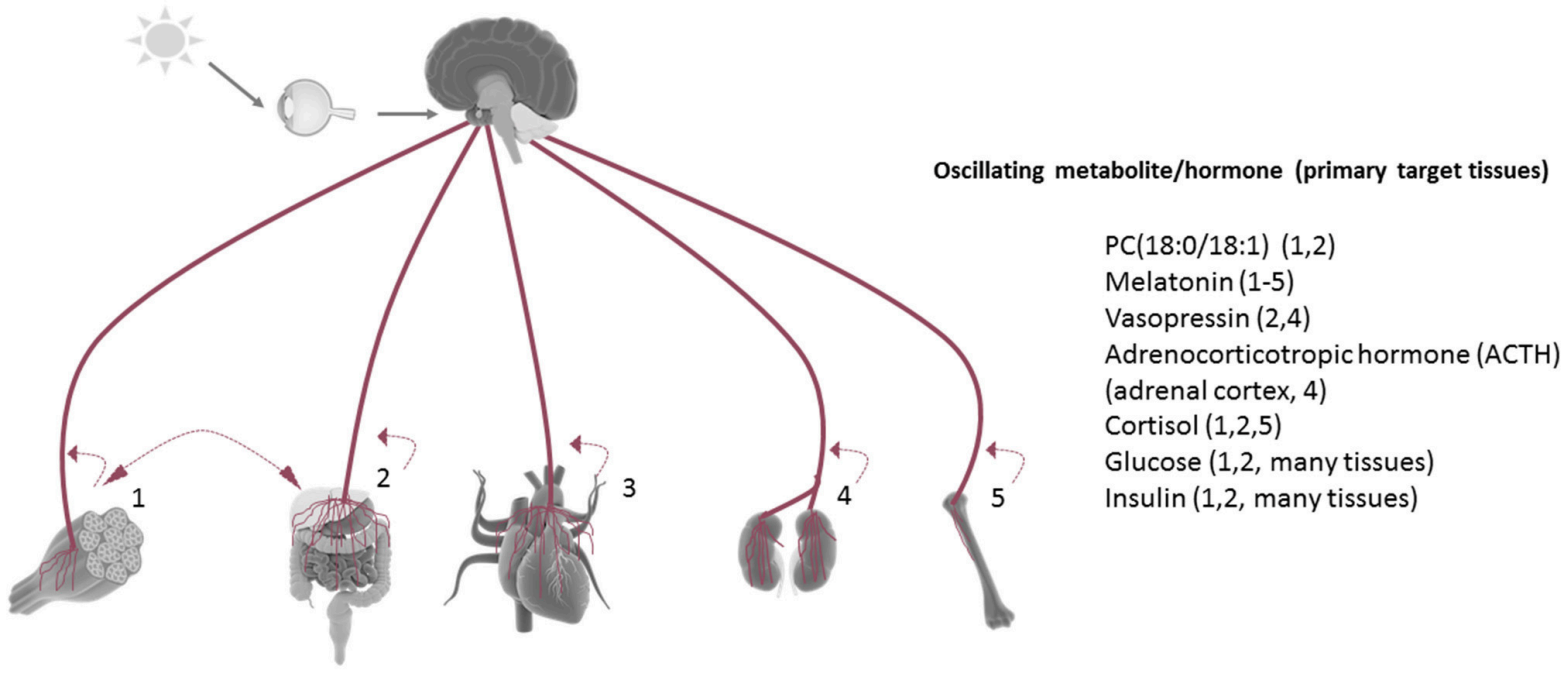
Tissue rhythmicity is in part driven by oscillating metabolites in the circulation. Some examples of rhythmic circulating metabolites (many of which are controlled by the central clock) which are linked to metabolic disruption when aberrantly released. Under nutrient challenge conditions, the majority of circadian metabolite oscillations in the serum are abolished. Most tissues release metabolites back into the circulation in a manner which reflects their circadian biological activities [examples: (1) activity/exercise, (2) nutrient metabolism, (3) myocardial contraction, (4) fluid filtration, (5) bone resorption]. Many tissue-derived circadian metabolites released in the circulation have been shown to directly influence the rhythmic energy use in another tissue (example: hepatic-derived PC18:0/18:1).

While numerous metabolites and hormones oscillate in a circadian manner *in vivo*, Figure 2 represents only a few of the many disparate rhythmic metabolites or hormones that orchestrate circadian responses within target tissues. For example, the phosphatidylcholine metabolites PC 18:0/18:1 have been shown to circulate in the serum as a result of rhythmic hepatic PPARδ activity, and in a manner which drives fatty acid use in the muscle (Liu et al., 2013). Interestingly, HFD reduces the circadian oscillation of this metabolite in the circulation. The dark-induced hormone melatonin is released by the pineal gland in a circadian fashion and targets many tissues via its rhythmic circulation and widespread expression of its receptors (Slominski et al., 2012). Interestingly, non-diabetic obese individuals have a significantly higher amplitude of melatonin oscillation compared to weight matched obese patients with type II diabetes, and lean non-diabetic individuals (Mantele et al., 2012). The antidiuretic hormone vasopressin is released in a circadian manner and has target effects on the liver (gluconeogenesis and glycogenolysis) and kidney, where it exerts its primary antidiuretic effects. Genetic variance in the vasopressin receptor AVPR1B have recently been linked to weight regulation, and copeptin, a C-terminal fragment of the arginine vasopressin pro-hormone is predictive of diabetes mellitus and abdominal obesity (Enhorning et al., 2009, 2013). Adrenocorticotrophic hormone (ACTH) is released rhythmically by the pineal gland, yet antiphase to melatonin. Subsequent stimulation of the adrenal cortex results in rhythmic cortisol release in humans as well as rhythmicity in aldosterone production and release. Cushing's disease, characterized by an overproduction of ACTH by the pituitary, severely diminishes the amplitude of oscillating cortisol in human patients (Boyar et al., 1979). Glucose and insulin, both elevated in the context of metabolic disease, are also present in the circulation in a rhythmic fashion, and directly depend on the circadian clock (Marcheva et al., 2011; Kalsbeek et al., 2014).

## Conclusions and future directions

Circadian metabolomics is just beginning to shed light on some of the mechanisms underlying our circadian behavior and physiology, and much more remains to be gained by such experiments. The opportunity to discover new biomarkers, to better predict physiological time, and to develop novel insights for personalized medicine are key areas for which circadian metabolomics experiments are likely to be extremely valuable in the near future.

### New biomarkers

As the number of published circadian metabolomics experiments continues to increase, one hope is that these uniquely robust and rich datasets will help serve to uncover novel biomarkers or complex metabolic signatures of various diseases. For example, jet-lag paradigms in mice have recently been shown to induce hepatocellular carcinoma (HCC), a phenomenon that appears to depend at least in part on impaired circadian metabolism in the liver (Kettner et al., 2016). Interestingly, jet-lag and mouse models of circadian disruption share this predisposition for HCC, as well as the altered circadian profile of numerous serum and hepatic carnitines, lipids and prostaglandins, TCA metabolites, and acetyl-CoA metabolites. Other studies looking at lung tumor-bearing mice also reveal altered circadian metabolite signatures (Masri et al., 2016). If circadian neuroendocrine and metabolic markers could collectively or in isolation be an indication of predisposition to tumorigenesis, or alternatively as evidence of existing tumorigenesis, this would greatly assist in cancer prevention and treatment.

### Time prediction

One hope already envisioned by several groups is that a clear temporal mapping of gene and metabolite oscillations and interactions will help us to better predict internal time. For example, can we predict drug efficacy or toxicity based on metabolite-based chronotype determinations? Can we determine the time of death? Blood metabolomics in mice has been studied for the purpose of predicting internal time (Minami et al., 2009) and metabolomics performed by breath analysis, where “breathprinting” is accomplished over hourly intervals, has revealed the potential for circadian rhythms in a large number of metabolites (Martinez-Lozano Sinues et al., 2014). Such an approach could greatly assist with chronotype determination. As the metabolomes of more subjects are studied with greater resolution, and better categorized into specific health and disease states, time prediction is likely to be a powerful end point of circadian metabolomics data for a variety of research areas.

A potentially fruitful outcome for circadian metabolomics as it relates to time prediction is its use in chronotherapy. Pharmacometabolomics is used to understand drug pharmacokinetics and pharmacodynamics. However, a better understanding of circadian fluctuations throughout the day and under specific environmental conditions could facilitate determination of optimal drug administration times, considering the toxicity and efficacy achieved in the body by the drug. Some drugs have already been shown to be more effective and/or less toxic when taken at a specific time of the day. One example is 5-fluorouracil (5-FU), which targets the oscillating thymidylate synthase, and which is already exploited for its effectiveness when administered at specific circadian times (Levi et al., 1992), when its toxicity is lowest at the level of bone marrow and gut, while concomitantly having the most antitumor effects (Wood et al., 2006). A recent high-throughput analysis importantly stressed that many more widely used drugs remain to be studied in this context (Zhang et al., 2014). Accordingly, personalized chronotherapy will undoubtedly emerge as a critical component of personalized medicine's future, as it holds great promise for disease treatment (Ballesta et al., 2017). It is hoped that additional knowledge gained from existing and future circadian metabolomics studies will facilitate and expedite our progression into this future.

## Author contributions

All authors listed, have made substantial, direct and intellectual contribution to the work, and approved it for publication.

### Conflict of interest statement

The authors declare that the research was conducted in the absence of any commercial or financial relationships that could be construed as a potential conflict of interest.
